# Investigation on the structures and magnetic properties of carbon or nitrogen doped cobalt ferrite nanoparticles

**DOI:** 10.1038/s41598-018-26341-4

**Published:** 2018-05-21

**Authors:** Derang Cao, Lining Pan, Jianan Li, Xiaohong Cheng, Zhong Zhao, Jie Xu, Qiang Li, Xia Wang, Shandong Li, Jianbo Wang, Qingfang Liu

**Affiliations:** 1 College of Physics, Laboratory of Fiber Materials and Modern Textile, the Growing Base for State Key Laboratory, Qingdao University, Qingdao, 266071 China; 2 Key Laboratory for Magnetism and Magnetic Materials of the Ministry of Education, Lanzhou University, Lanzhou, 730000 China

## Abstract

Carbon or nitrogen doped cobalt ferrite nanoparticles were synthesized in the air by a facile calcination process. X-ray diffraction, mapping, X-ray photoelectron spectroscopy, and mössbauer spectra results indicate that the nonmetal elements as the interstitial one are doped into cobalt ferrite nanoparticles. The morphologies of doped cobalt ferrite nanoparticles change from near-spherical to irregular cubelike shapes gradually with the increased carbon or nitrogen concentration, and their particles sizes also increase more than 200 nm. Furthermore, the saturation magnetization of carbon doped cobalt ferrite is improved. Although the saturation magnetization of N-doped cobalt ferrite is not enhanced obviously due to the involved hematite, they also do not drop drastically. The results reveal an approach to synthesize large scale ferrite nanoparticles, and improve the magnetic properties of ferrite nanoparticles, and also provide the potential candidates to synthesis co-doped functional magnetic materials.

## Introduction

As is well-known, doping is a vitally common and important approach to modify the structures and properties of the materials by changing the ions distribution during the synthesis process^1–8^. The doping materials present the satisfying properties for lots of advanced applications such as battery^9^, capacitance^10^, electronic device^11^, catalysis^12^, etc. Lots of these properties and applications recently show a close relation with magnetic nanoparticles^3,6,13–15^, which requires the good magnetism of materials. Large magnetization, suitable particle size, and good dispersibility of the magnetic nanoparticles are desirable for the magnetic labels of biodetection^16^. However, the magnetization of the particles with smaller size (usually less than 20 nm) is less than that of the microbeads and the submicro or nanoparticles^17^. This work demonstrated a facile approach to synthesize large scale ferrite nanoparticles, and improved the magnetic properties of nanoparticles.

Generally, both metal and nonmetal elements are the normal origin of doping elements. The metal elements are more inclined to be doped into many magnetic materials^3,4,6,18–23^ and some nonmagnetic materials^10,12,24,25^, while the nonmetal elements (like carbon, nitrogen, phosphor, and sulfur, etc.) usually focus on the doping of nonmagnetic materials, such as titanium dioxide^26,27^, carbon materials^28,29^, silicon materials^30,31^ and other nonmagnetic materials^11,24,32^. This is due to the doped nonmetal elements can influence the electronic mobility or the band gap of crystal^30,33,34^, and thus improves the properties of these nonmagnetic materials. However, it is more inclined to be doped with the metal elements for the magnetic materials^35–43^, and these results only present in the theory researches^44,45^ and a small number in experimental magnetic films^46–50^, which are prepared by vacuum deposition^46–50^ or chemical vapor deposition^51,52^. The reason is that it is difficult for the nonmetal elements to be doped into the lattice of magnetic films^53^, and they always lies in the gap or the edge of the atom as a interstitial one, which is easy to achieve doping under vacuum condition^46–50^. Whereas, the metal elements can change the site preference of magnetic materials, and locate the lattice of magnetic materials, and further influence the magnetic properties of the samples^35,54,55^. As a result, the reports on the doping of nonmetal elements into magnetic nanoparticles are infrequent, and it should be considered.

Cobalt ferrite (CoFe_2_O_4_), as a particularly important magnetic material, owns large magneto-crystalline anisotropy, high coercivity, large magnetostriction coefficient, and high saturation magnetization (*M*_s_), and it has attracted the considerable attention for a long time^56–59^. Numerous investigations on the doping of CoFe_2_O_4_ nanoparticles have been reported including their structures and magnetic properties^3–5,19,21,39,40,42,60–65^. As mentioned above, nevertheless, the main studies of CoFe_2_O_4_ nanoparticles also focused on the doping of metal elements (i.e. Li^19^, Mn^21^, Zn^66^, Ni^67^, Ga^62^, Dy^60^, In^40^, Bi^4^, Gd^68^, Cr^69^, Er^39^, Ho^63^, Ti^70^, La^56^, Ce^36^, RE^55,71,72^, etc.). These metal elements always substitute Fe or Co ions, and locate the lattice of CoFe_2_O_4_ nanoparticles. The *M*_s_ of CoFe_2_O_4_ nanoparticles often decreases after the doping due to the smaller magnetic moments of doped metal elements than that of the Fe or Co ions, and it is unsuitable for high *M*_s_ applications. There are only a few works showing the improvement of *M*_s_ after doping with metal elements^4,21,69^. The changed *M*_s_ are mainly affected by the site preference of tetrahedral (A) and octahedral (B) of Fe or Co ions. Saturation magnetization *M*_s_ is equal to the difference of the magnetizations of the two sites, i.e., *M*_s_ = (*μ*_B_ − *μ*_A_), where *μ*_B_ and *μ*_A_ are the magnetizations of B and A sites respectively^21^. Until now, reports regarding the structures and magnetic properties of nonmetal doped CoFe_2_O_4_ nanoparticles are less. Our recent work^5^ studied the results of sulfur doped CoFe_2_O_4_ nanoparticles, and the results showed an enhanced *M*_s_ after the doping. However, carbon or nitrogen doped CoFe_2_O_4_ nanoparticles have not been demonstrated, and their results are still unclear.

In this work, series of nonmetal carbon or nitrogen doped CoFe_2_O_4_ particles were prepared in the air via a facile calcination process, and this approach is different from the previous vacuum deposition method^46–50^ and chemical vapor deposition^51,52^ technique in the doping of nonmetal elements. There are no *p*H regulation, gas atmosphere, centrifugation and any another supplementary reagents during the preparing process. Various characterizations showed that carbon or nitrogen contents were doped into CoFe_2_O_4_ nanoparticles, and the *M*_s_ of doped CoFe_2_O_4_ nanoparticles was improved with the increased doping concentration. Combining our previous S-doped CoFe_2_O_4_ nanoparticles^5^, the results reveal an approach to improve the magnetic properties of ferrite nanoparticles.

## Experimental

**Figure 1:**
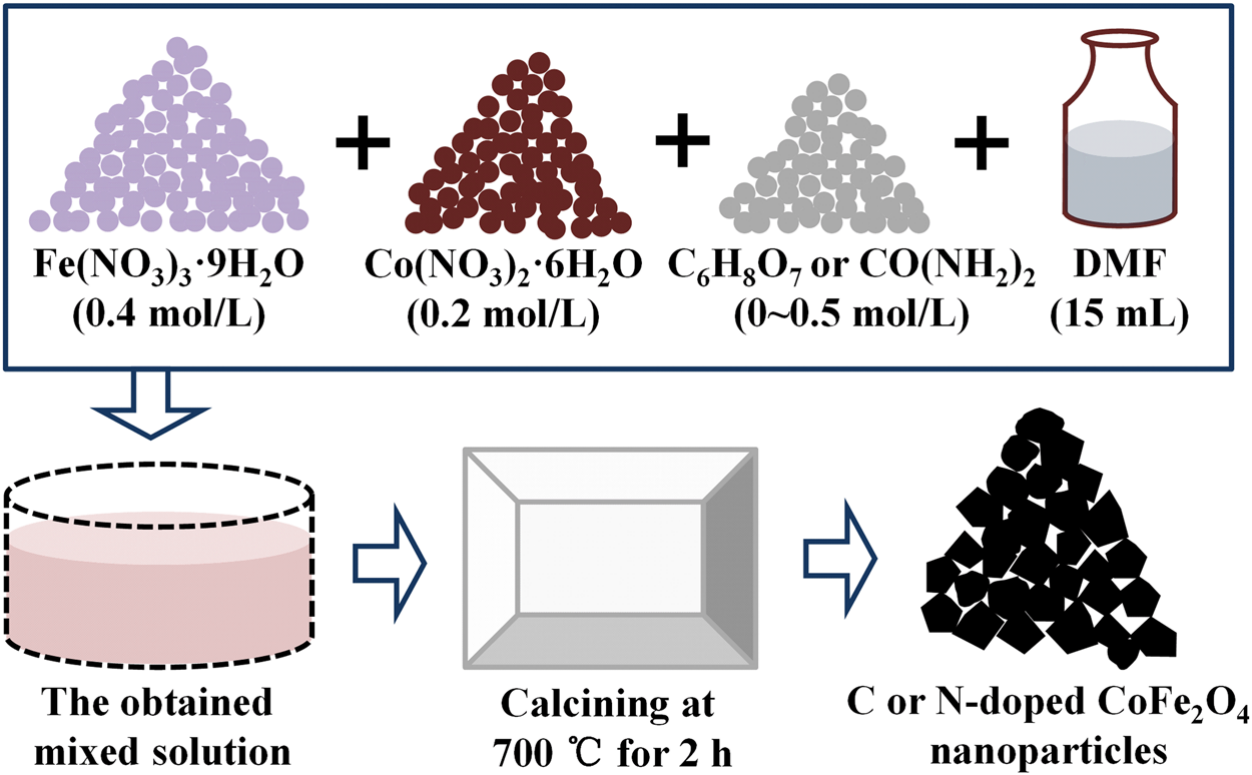
Schematic diagram of the simple experimental process.

Ferric nitrate (0.4 mol·L^−1^), cobalt nitrate (0.2 mol·L^−1^) and various concentrations of citric acid (or urea) were dissolved in Dimethyl Formamide (DMF, 15 mL). The synthesis process can be simply described as Fe(NO_3_)_3_ + Co(NO_3_)_2_ + C_6_H_8_O_7_ (or CO(NH_2_)_2_) + DMF + calcination → C or N doped CoFe_2_O_4_, and the schematic diagram of the simple experimental process is shown in Fig. 1. The citric acid and urea were the origin of doped C and N elements, and the concentrations were 0 mol·L^−1^, 0.05 mol·L^−1^, 0.1 mol·L^−1^, 0.18 mol·L^−1^, 0.3 mol·L^−1^, and 0.5 mol·L^−1^ respectively. The obtained mix solution was calcined at 700 °C for 2 hour in air, and the heating rate was 1 °C/min. The obtained products were the C or N doped CoFe_2_O_4_ nanoparticles.

The structure of nanoparticle was measured by X-ray diffraction (XRD, PANalytical X’Pert) equipped with Cu-Kα radiation (λ = 1.5406 Å). The morphology of sample was observed by using transmission electron microscopy (TEM, Tecnai^TM^ G^2^ F30, FEI) and field emission scanning electron microscopy (SEM, Hitachi S-4800) equipped with an energy-dispersive spectrometer. The X-ray photoelectron spectroscopy (XPS, PHI-5702, Physical Electronics) was performed using a monochromatic Mg-K_α_ irradiation and a charge neutralizer, and all binding energies were referred to the C1s peak at 284.6 eV of the surface adventitious carbon. Mössbauer spectra were recorded at room temperature using a conventional constant acceleration spectrometer with a γ-ray source of 25 mCi ^57^Co in a palladium matrix. The magnetic properties of the samples were measured using a vibrating sample magnetometer (VSM, Lakeshore 7304).

## Results and Discussion

**Figure 2:**
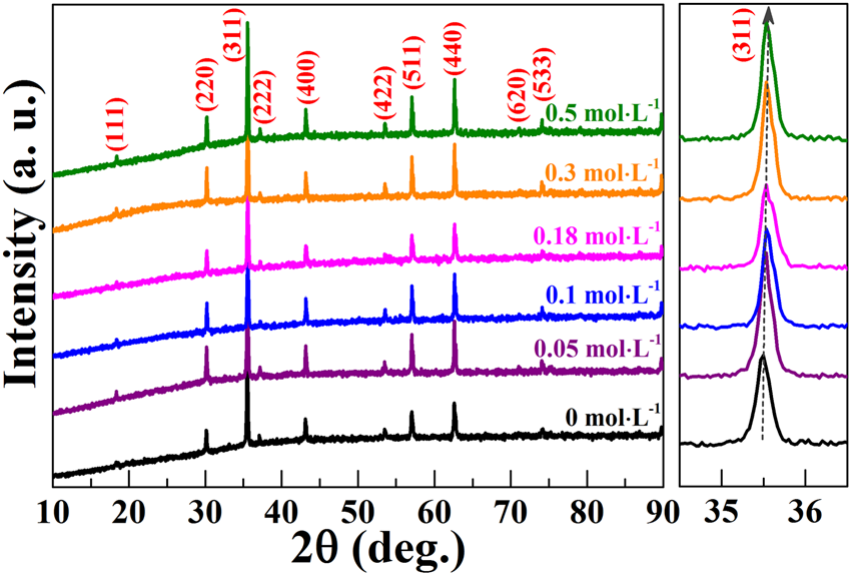
XRD patterns for all C-doped CoFe_2_O_4_ samples with different citric acid concentrations.

The structures for all C-doped CoFe_2_O_4_ samples with different citric acid concentration were performed by XRD, which is shown in Fig. 2. It can be seen that the samples show good single CoFe_2_O_4_ phase with the cubic lattice (JCPDS#22-1086) when the concentration of citric acid increases from 0 mol·L^−1^ to 0.5 mol·L^−1^, and all the diffraction peaks are well indexed. The diffraction peaks (311) of the samples shift to the higher angles as citric acid concentration increases to 0.18 mol·L^−1^, and then almost keep unchanged. The results suggeste a reduction of lattice with the improvement of citric acid concentration, which reveals the influence of additional citric acid on the structure of samples. Previous studies for the synthesis of zirconia showed the same variation with the improvement of citric acid content^73,74^. When urea is doped into CoFe_2_O_4_ samples, the XRD patterns for N-doped CoFe_2_O_4_ samples with different urea concentration are shown in Figure S1. It can be observed that the diffraction peaks (104) of Fe_2_O_3_ are also observed except for the CoFe_2_O_4_ phases when urea concentration increases from 0 mol·L^−1^ to 0.5 mol·L^−1^. The main diffraction peaks (311) of CoFe_2_O_4_ shifts to the higher angles as urea concentration increases to 0.1 mol·L^−1^, and then the peak keeps almost unchanged when the secondary phase Fe_2_O_3_ exists in particles. The results indicate that the additional urea can affect the structure of CoFe_2_O_4_ nanoparticles, but it will also introduce other impurities of Fe_2_O_3_ when urea concentration is high. Previous works also showed that the excess of urea could cause additional hematite Fe_2_O_3_ phase in ferrite nanoparticles^75,76^, and the gases generated by urea are easily released at the moment, which is adverse for the crystallization of ferrite^75–78^. Thus, the hematite Fe_2_O_3_ phase is generated with the improvement of urea concentration.

**Figure 3:**
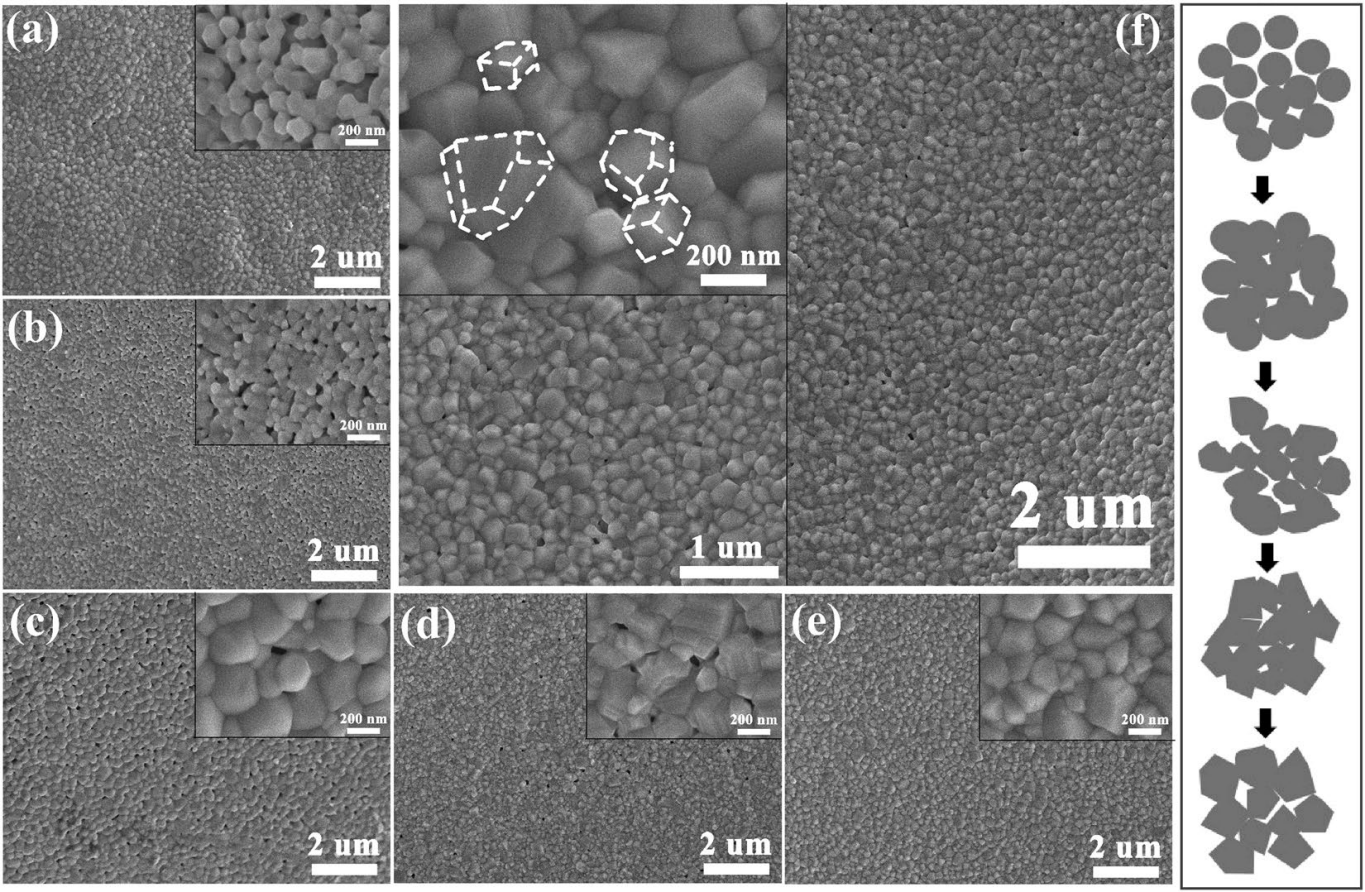
SEM imagines for all C-doped CoFe_2_O_4_ samples with different citric acid concentration: (**a**) 0 mol·L^−1^, (**b**) 0.05 mol·L^−1^, (**c**) 0.1 mol·L^−1^, (**d**) 0.18 mol·L^−1^, (**e**) 0.3 mol·L^−1^, and (**f**) 0.5 mol·L^−1^, respectively. The inset in each picture is the amplifying results. The right picture is the simply formation process of the samples with the increase of citric acid concentration.

Typical SEM images for all C-doped CoFe_2_O_4_ samples with different citric acid concentration are shown in Fig. 3. Pure CoFe_2_O_4_ nanoparticles showed in Fig. 3(a) are composed of a large quantity of uniform microspheres. The CoFe_2_O_4_ particles begin to reunite together when citric acid is introduced to precursor (Fig. 3b), and the samples are composed of large and agglomerative particles which are surrounded by lots of small nanoparticles. As citric acid content is further increased (Fig. 3c), obvious smooth and compact nanoparticles can be seen, and the particle sizes increase. These results reveal that CoFe_2_O_4_ nanoparticles are further grown when citric acid is added during the synthetic process, and the particles increase with the improvement of citric acid concentration. Afterwards, CoFe_2_O_4_ particles begin to reunite together when citric acid concentration is 0.18 mol·L^−1^ (Fig. 3d), and the samples change from near-spherical to irregular cubelike shapes. Then CoFe_2_O_4_ nanoparticles become compact and irregular cubelike shapes with the further improvement of citric acid concentration, which are shown in Fig. 3(e,f). The average particles size is more than 200 nm when citric acid concentration is 0.18 mol·L^−1^. These formation process with the increase of citric acid concentration is simply described by the right picture of Fig. 3. In addition, it can be seen from SEM results that the particles size seems to increase with the improvement of citric acid concentration. The particle sizes obtained from SEM are not accurate due to the aggregation of the samples, and it is not shown here. One can deduce that the particles size increases and the shape changes to irregular cubelike shapes after the doping of C element. It is reported that the increasing citric acid contents can improve the degree of chelation of metallic ions in the solution, which results in a higher uniformity of metallic ions during the synthetic process^79,80^. It is also reported that the increasing concentration of citric acid can reduce the formation temperature of ferrite^81^, in other words, the ferrites are easier formed when the citric acid is added under the same calcination temperature. In this work, when citric acid is added in the precursors, the uniformity and dispersion of Fe and Co ions become better, the reactions of solution are more sufficient, and the local calcination temperature around ferrite molecule may be improved. These conditions could enhance the growth rate of particles, and the particle sizes increase. The growth rate of the particle increases faster with the further improvement of citric acid, and then results in the change of nanoparticle shape. It has been demonstrated that the shape of the nanoparticles can also be reversibly interchanged between spherical and cubic shape by controlling particles growth rate^57^. The SEM images for N-doped CoFe_2_O_4_ samples with different urea concentration are similar to the results of C-doped CoFe_2_O_4_ nanoparticles, which are shown in Figure S2.

**Figure 4:**
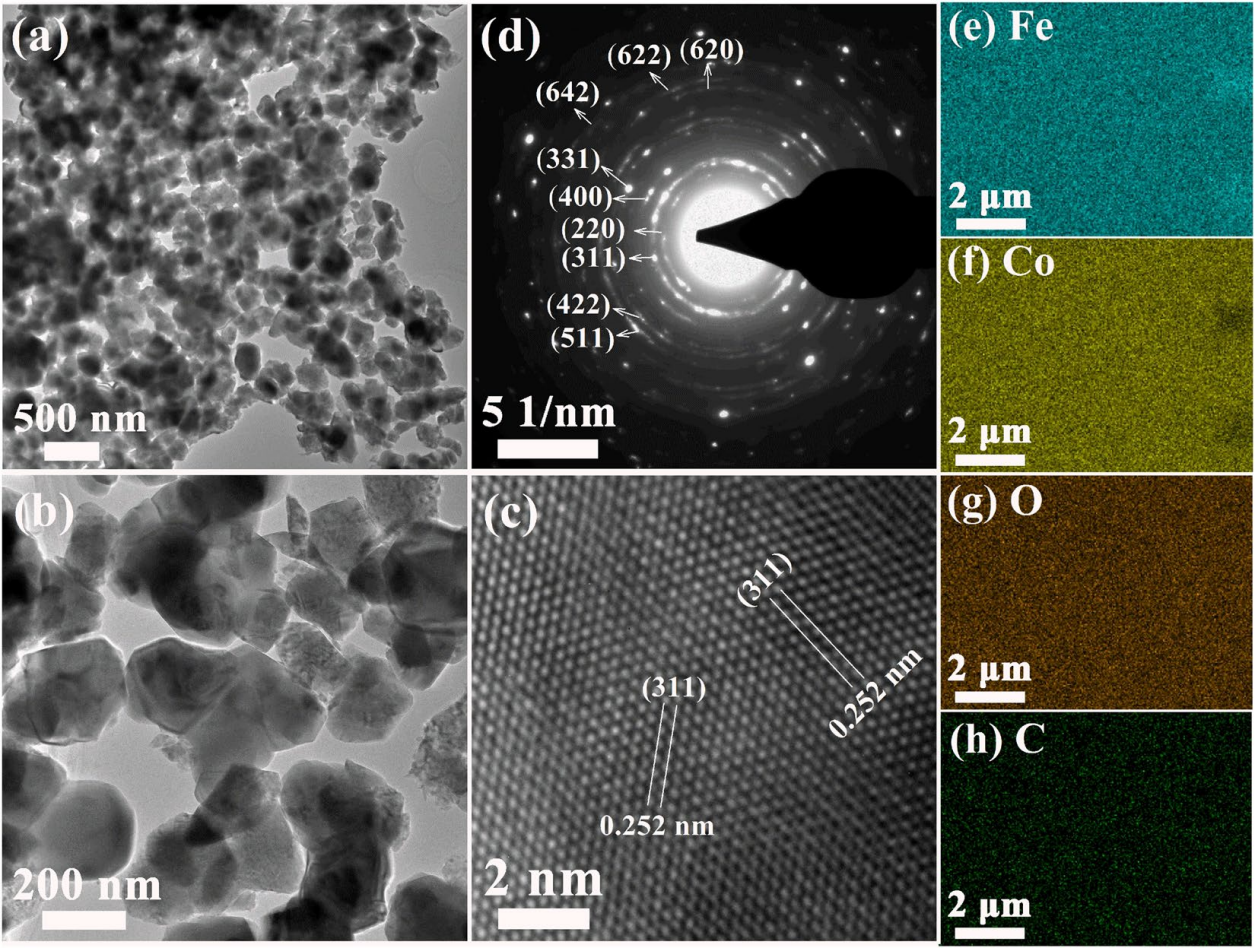
C-doped CoFe_2_O_4_ sample with citric acid concentration of 0.5 mol·L^−1^. (**a**,**b**) Typical TEM images, (**c**) HRTEM image, (**d**) SAED; Elemental mappings: (**e**) Fe element; (**f**) Co element; (**g**) O element, and (**h**) C element.

As a representative, the morphology and structure for C-doped CoFe_2_O_4_ with the citric acid concentration of 0.5 mol·L^−1^ are further characterized by TEM. As shown in Fig. 4(a,b), the results indicate large and black areas of irregular cubelike CoFe_2_O_4_ nanoparticles, and the average particles size is about 220 nm. HRTEM characterizations present the lattice fringes of the sample, and the interfringe distance shown in Fig. 4(c) is 0.252 nm, which is correspond well to {311} planar spaces of CoFe_2_O_4_ nanoparticles. The SAED (Fig. 4d) clearly presents a group of atomic planes of the particles, revealing the highly crystalline nature of these nanoparticles. A large number of C-doped CoFe_2_O_4_ nanoparticles with the citric acid concentration of 0.5 mol·L^−1^ are used to investigate the elemental distribution by using EDX mapping measurement. The impurities in the electron microscopy, e.g. carbon coated grids, has been avoided and excluded during the sample preparation to obtain an accurate results. As shown in Fig. 4(e–h), all Fe, Co, O and C elements distribute evenly and uniformly throughout the CoFe_2_O_4_ nanoparticles, which confirm that C has been incorporated into the CoFe_2_O_4_ nanoparticles. However, the mapping of C element does not display very obvious profile when compared to others, which can be concluded that a few C elements are incorporated into the nanoparticles. The EDX mapping measurement of N-doped CoFe_2_O_4_ samples (0.18 mol·L^−1^) shown in Figure S2 also indicates that a few N contents are incorporated into CoFe_2_O_4_ nanoparticles.

**Figure 5:**
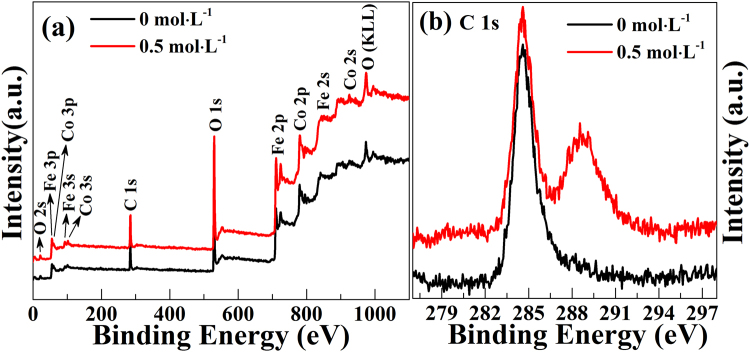
XPS data for the pure CoFe_2_O_4_ (0 mol·L^−1^) and C-doped CoFe_2_O_4_ samples with citric acid (0.5 mol·L^−1^): (**a**) the full scan, (**b**) C 1 s level.

The elemental composition is examined by XPS to further distinguish the chemical composition of C-doped CoFe_2_O_4_ nanoparticles, which is displayed in Fig. 5(a) shows the full scanned XPS spectra of the pure CoFe_2_O_4_ (0 mol·L^−1^) and C-doped CoFe_2_O_4_ (0.5 mol·L^−1^) samples. It can be seen that Co 2p, Fe 2p, O 1 s and C 1 s peaks appear in the pure CoFe_2_O_4_ and C-doped CoFe_2_O_4_, and the full scan spectra of two samples show no obvious change. Figure 5(b) presents the C 1 s core-level XPS spectra of two samples recorded in the high-resolution mode. It can be observed that one high peak is detected at about 284.6 eV, which belongs to the carbon contaminants absorbed on the surface of the tested samples. However, C-doped sample also exhibits another obvious peak at about 288.8 eV, and this major contribution is assigned to C and O species^82–85^. As well known, the C atom is very small, which is difficult to substitute for the ions of CoFe_2_O_4_, and the XPS peak or spoor of C element in this work is not found at about 162 eV, indicating the absence of anionic carbon, i.e., there cannot exist the substitution between C and O elements^86,87^. The C element may be a interstitial one which is absorbed near the gap or edge of CoFe_2_O_4_^88^. The XPS data further prove that C element has been incorporated into CoFe_2_O_4_ nanoparticles, which agrees well with XRD spectra and EDX mapping results.

**Figure 6:**
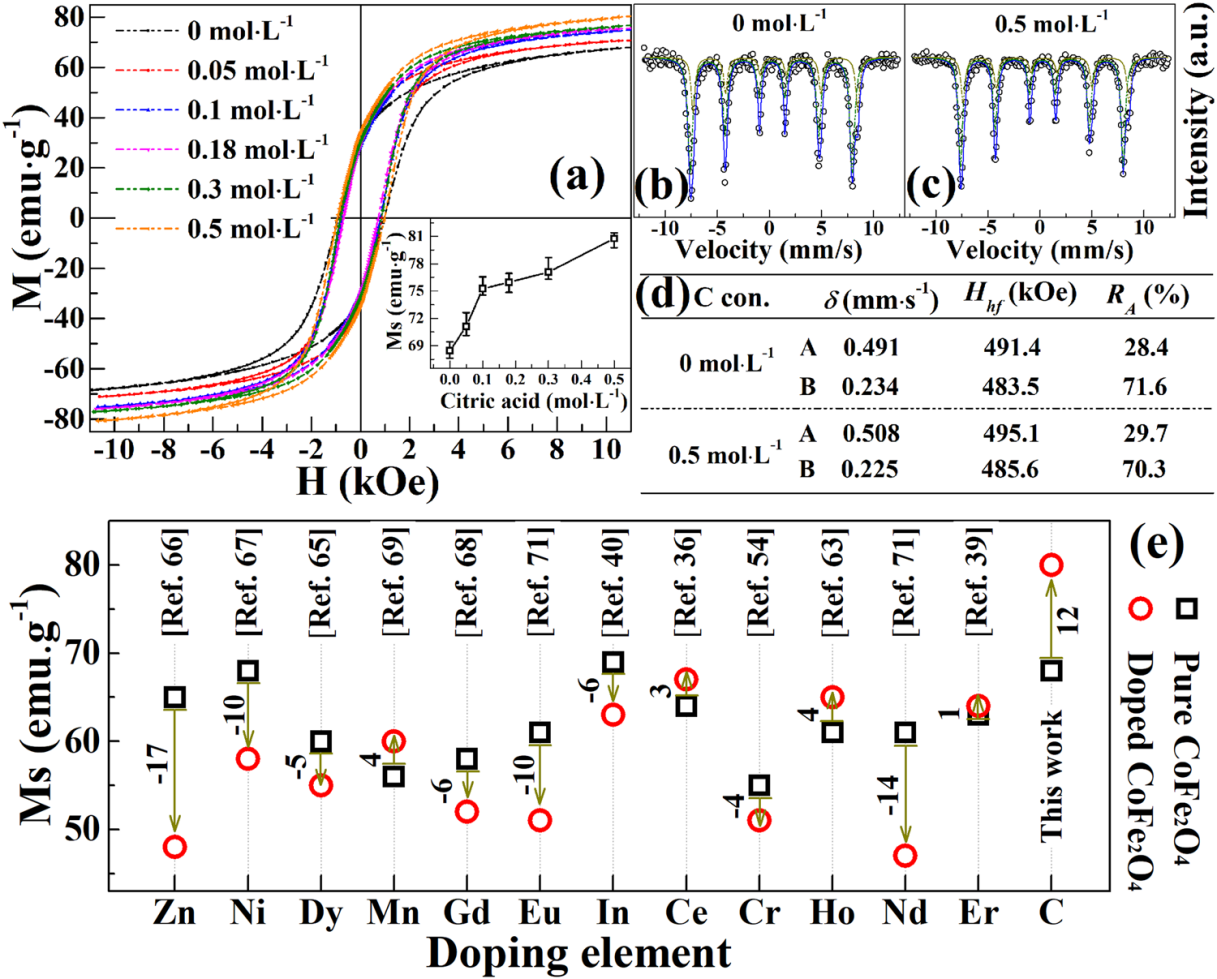
(**a**) *M-H* loops for C-doped CoFe_2_O_4_ nanoparticles with different citric acid concentration; the inset is citric acid concentration dependence of *M*_s_ for the corresponding samples. (**b**,**c**) Mössbauer spectra for the pure CoFe_2_O_4_ (0 mol·L^−1^) and C-doped CoFe_2_O_4_ nanoparticles (0.5 mol·L^−1^). (**d**) Mössbauer parameters for two samples obtained from the mössbauer spectra. (**e**) Comparison of our work and other typical CoFe_2_O_4_ nanoparticles for the variability of *M*_s_ after doping.

The room temperature magnetic performances of products are discussed as below base on the understanding of the microstructure and chemical phase. As shown in Fig. 6(a), the magnetic hysteresis (*M-H*) loops reveal that all C-doped CoFe_2_O_4_ samples perform a typical ferromagnetic property at room temperature. The magnetic saturation (*M*_s_) is about 68 emu·g^−1^ for the pure CoFe_2_O_4_, which is close to the theoretical values of 71.2 emu·g^−1^ ^89^. The *M*_s_ of CoFe_2_O_4_ nanoparticles monotonously increases with the improvement of citric acid concentration. The inset of Fig. 6(a) exhibits the citric acid concentration dependence of *M*_s_ of all samples. It can be seen when citric acid concentration reaches to 0.5 mol·L^−1^, the value of *M*_s_ increases to 80 emu·g^−1^, which is much larger than previously reported CoFe_2_O_4_ nanoparticles^39,40,54,55,65,69,71,89^. Significantly, C-doped CoFe_2_O_4_ nanoparticles in this work are quite different from the former metal-doped CoFe_2_O_4_ nanoparticles, which mainly show a decreased *M*_s_ after doping, but the *M*_s_ is improved greatly in this work. Comparisons of the variability of *M*_s_ after doping are shown in Fig. 6(e). The results are consistent with our previous S-doped CoFe_2_O_4_ nanoparticles^5^, which also showed an increasing *M*_s_ after the doping of S element. Both of the results reveal an approach to synthesize large scale ferrite nanoparticles, and improve the magnetic properties of ferrite nanoparticles.

Mössbauer spectroscopy gives information of the spatial orientation of a magnetic sublattice with respect to its net magnetization^90^, and the spectra for the pure CoFe_2_O_4_ (0 mol·L^−1^) and C-doped CoFe_2_O_4_ sample (0.5 mol·L^−1^) are shown in Fig. 6(b,c). The results show well-resolved two sextets due to Fe^3+^ at the tetrahedral (A) sites and another Fe^3+^ at octahedral (B) sites, and it presents a typical spinel structure reported by other authors^56,62^. The mössbauer parameters for two samples, i.e., hyperfine field (*H*_*hf*_) and isomer shift (*δ*) obtained from the mössbauer spectra are demonstrated in Fig. 6(d). The value of *δ* at A site of C-doped CoFe_2_O_4_ is decreased while it is increased at the B site, and this depends on the s-electron charged density of the absorber^60^. In addition, a little bigger *H*_*hf*_ of C-doped CoFe_2_O_4_ is observed when comparing to pure CoFe_2_O_4_. These results indicate that Co^2+^ ions migrates from B site to A site and hence Fe^3+^ ions changes from A site to B site after doping of C element. As well known, the increasement of *M*_s_ can be achieved by either increasing of moments at site B and decreasing of moments at site A or both^21^. However, the changed site of A and B in this work is not the main reason for the improvment of *M*_s_. The obtained values of mössbauer parameters for the samples are very close, and thier little differences may mainly come from the calculated errors. Combining the results of previous S-doped CoFe_2_O_4_^5^ and this work, the enhanced *M*_s_ of C-doped CoFe_2_O_4_ nanoparticles is related to the improvement of the particle size and the change of particle shape as well as their higher crystallinity of the nanoparticles with the increased citric acid concentration. As shown in SEM and TEM results, the enhancement of citric acid amount causes the nanoparticles to synthesize the big particle size and high crystallinity, which results in the high magnetization of C-doped CoFe_2_O_4_ nanoparticles. The enhanced *M*_s_ with the increase of nanoparticle size is similar to those results reported in other literature^91^, and it usually attributed to a decreasing proportion of the pinned surface magnetic moments in overall magnetization when the nanoparticle grows up in size^92,93^. In addition, previous research indicated the *M*_s_ of cubelike nanoparticles is larger than that of spherical nanoparticles^57^, and also demonstrated the higher crystallinity is benefited to enhanced the *M*_s_ of sample^94,95^. The flat surfaces of cubic nanoparticles enable the surface cations of metal to possess a more symmetric coordination, and the missing coordinating oxygen atoms is fewer when compared to the curved topologies of spherical nanoparticles. Therefore, the surface anisotropy should be much smaller in large cubelike nanoparticles, which would result in a smaller surface pinning and larger magnetization.

*M-H* loops for the N-doped CoFe_2_O_4_ samples nanoparticles with different urea concentration are shown in Figure S3, the results reveal that the *M*_s_ of N-doped CoFe_2_O_4_ nanoparticles has no obvious change, but decreases a little when compared to pure CoFe_2_O_4_. This is due to the presence of non-magnetic Fe_2_O_3_ according to XRD results, which leads to the relative reduction of magnetic CoFe_2_O_4_, and the total qualities of magnetic moments drop. The urea concentration dependence of *M*_s_ of all samples is shown in the inset of Figure S3. Significantly, the *M*_s_ of CoFe_2_O_4_ nanoparticles is not decreased sharply due to the non-magnetic Fe_2_O_3_, and there may exist two competitive relations in CoFe_2_O_4_ nanoparticles. The first one, i.e. non-magnetic Fe_2_O_3_, plays a role to reduce the *M*_s_ of CoFe_2_O_4_ nanoparticles, and the another one, i.e. the effect of doping element, promotes the improvement of the *M*_s_ of CoFe_2_O_4_ nanoparticles. As a result, the *M*_s_ of N-doped CoFe_2_O_4_ nanoparticles keeps a small fluctuation and does not increases or decreases sharply, which indirectly reflects that the *M*_s_ of CoFe_2_O_4_ nanoparticles can be enhanced after doping with N element.

## Conclusion

Nonmetal-doped CoFe_2_O_4_ nanoparticles were prepared via a facile calcination process in air. Characterizations of XRD, EDX mapping, XPS, and mössbauer spectra results confirm that the C or N element is doped into CoFe_2_O_4_ nanoparticles. The morphology of C or N doped CoFe_2_O_4_ nanoparticles changes from the microsphere to the irregular cubelike nanoparticles due to the reduced formation temperature of ferrite by the additional citric acid and urea, and their particles sizes also increase to more than 200 nm with the increasing citric acid or urea concentration. Particularly, the saturation magnetization of C-doped CoFe_2_O_4_ nanoparticles increase to 80 emu·g^−1^, but the saturation magnetization of N-doped CoFe_2_O_4_ has no obvious change due to the introduced non-magnetic Fe_2_O_3_. The results reveal an approach to synthesize large scale ferrite nanoparticles, and improve the magnetic properties of ferrite nanoparticles, and also provide the potential candidates to synthesize co-doped functional magnetic materials.

## Electronic supplementary material

Supplementary Information
