# Supplementary material for: Investigation on the structures and magnetic properties of carbon or nitrogen doped cobalt ferrite nanoparticles
Source: Sci Rep. 2018 May 21;8:7916. doi: 10.1038/s41598-018-26341-4 (PMC5962609; doi:10.1038/s41598-018-26341-4)
Supplement: Supplementary file 1 — Supplementary Information [file 41598_2018_26341_MOESM1_ESM.pdf]

Supplementary material

Investigation on the structures and magnetic properties of carbon or nitrogen doped cobalt ferrite nanoparticles

Derang Cao[a, b*], *Lining Pan*[b], *Jianan Li*[b], *Xiaohong Cheng*[b], *Zhong Zhao*[a], *Jie Xu*[a], *Qiang Li*[a], *Xia Wang*[a], *Shandong Li*[a], *Jianbo Wang*[b], *and Qingfang Liu*[b]

[a]College of Physics, Laboratory of Fiber Materials and Modern Textile, the Growing Base for State Key Laboratory, Qingdao University, Qingdao 266071, China

[b]Key Laboratory for Magnetism and Magnetic Materials of the Ministry of Education, Lanzhou University, Lanzhou 730000, China

*Corresponding author: caoderang@163.com

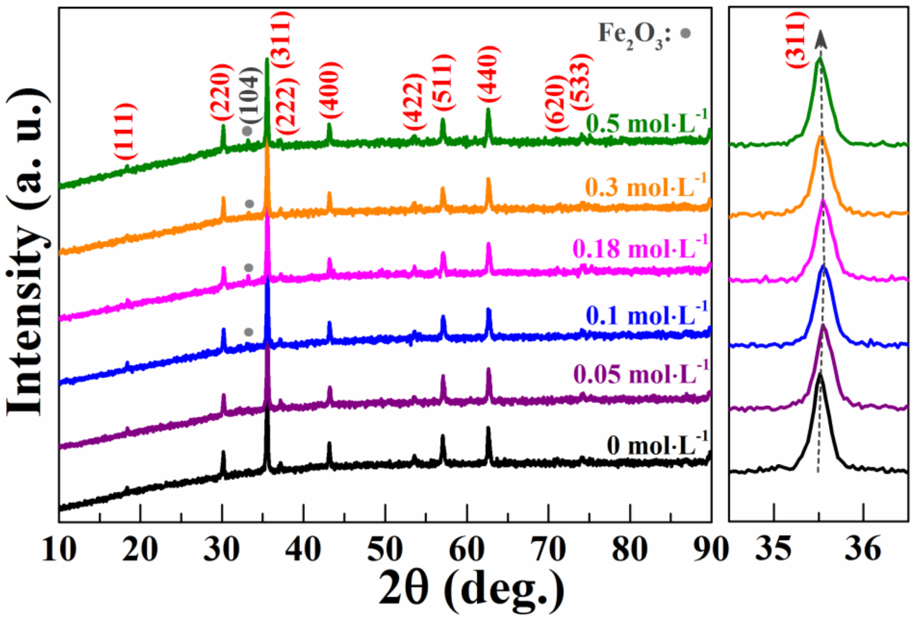

Figure S1 XRD patterns for all N-doped $CoFe_2O_4$ samples with different urea concentration.

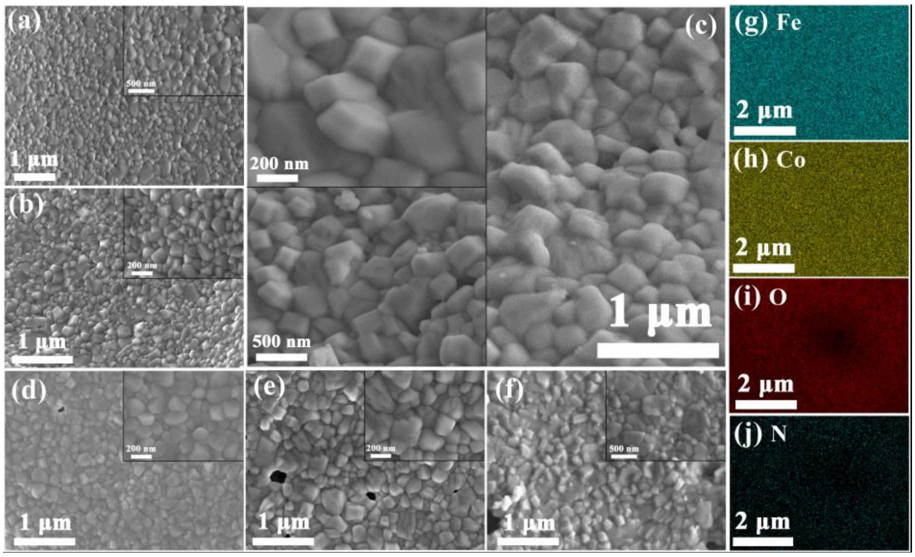

Figure S2 SEM imagines for N-doped $CoFe_2O_4$ samples with different urea contents: (a) 0 mol·L^{-1}, (b) 0.05 mol·L^{-1}, (c) 0.1 mol·L^{-1}, (d) 0.18 mol·L^{-1}, (e) 0.3 mol·L^{-1}, and (f) 0.5 mol·L^{-1}, respectively. The inset in each picture is the amplifying results. Elemental mappings for N-doped $CoFe_2O_4$ with the urea concentration of 0.18 mol·L^{-1}: (e) Fe element; (f) Co element; (g) O element, and (h) C element.

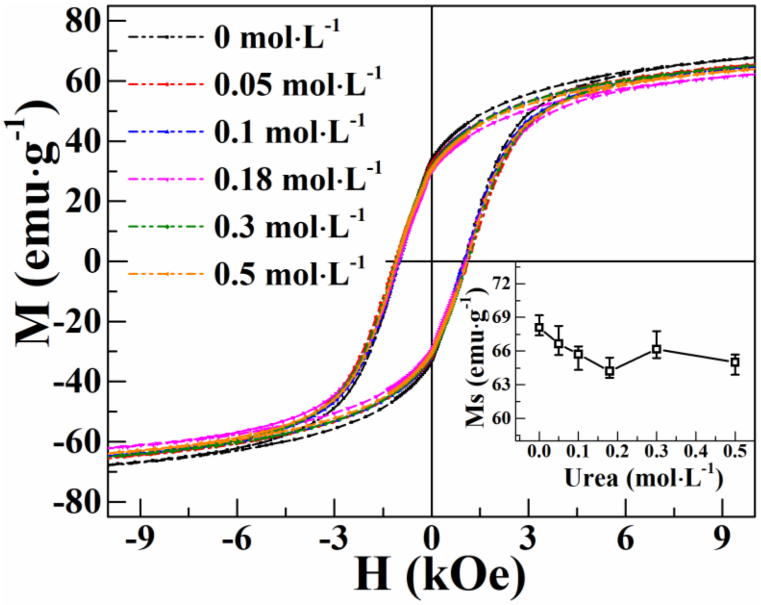

Figure S3 *M-H* loops for N-doped $CoFe_2O_4$ nanoparticles with different urea concentration; the inset is urea concentration dependence of M_s for the corresponding samples.
